# Effectiveness of a gamified educational intervention on hand hygiene among Nursing professionals: a quasi-experimental study

**DOI:** 10.1590/1518-8345.8067.4891

**Published:** 2026-08-10

**Authors:** Maiara Luci Silva Costa, Márcio Medeiros, Fábio da Costa Carbogim, Thiago César Nascimento, André Luiz Silva Alvim

**Affiliations:** 1 Universidade Federal de Juiz de Fora, Juiz de Fora, MG, Brazil.; 2 Universidade Federal de Juiz de Fora, Faculdade de Enfermagem, Juiz de Fora, MG, Brazil.; 3 Universidade Federal de Juiz de Fora, Faculdade de Enfermagem, Departamento de Enfermagem Aplicada, Juiz de Fora, MG, Brazil.; 4 Universidade Federal de Juiz de Fora, Faculdade de Enfermagem, Departamento de Enfermagem Básica, Juiz de Fora, MG, Brazil.

**Keywords:** Hand Disinfection, Nursing Team, Gamification, Health Education, Education, Infection Control.

## Abstract

**Objective:**

to assess the effectiveness of a gamified educational intervention on proper execution of the hand hygiene technique among nurses and nursing technicians in hospitalization units.

**Method:**

this quasi-experimental study of the before-and-after type was conducted with 45 Nursing professionals. The data were collected by means of a structured form to observe the technique before and after the intervention. The statistical data analysis was performed using Wilcoxon’s Signed-Rank test and the McNemar test.

**Results:**

in the pre-intervention period, 61.3% of the technicians and 42.9% of the nurses failed to follow all the technique steps; after the gamification, 100% of the nurses and 97.7% of the nursing technicians did implement the right steps (*p*<0.001) and forgot fewer stages (*z*=-4.532, *p*<0.001, *r*=0.81). The time to perform the technique was also adequate after the intervention, following the recommendations set forth by the World Health Organization (*z*=-2.441, *p*=0.015, *r*=0.36).

**Conclusion:**

the gamified educational intervention was effective in proper execution of the hand hygiene technique among nurses and nursing technicians, reducing errors and increasing compliance with the international guidelines.

## Highlights

**(1)** Gamification increased adherence to hand hygiene among Nursing professionals. **(2)** Gamification proved to exert a positive impact on learning and adherence to the technique. **(3)** There was a significant reduction in the number of stages forgotten during hand hygiene procedures. **(4)** Product distribution on the hands was more uniform after the intervention. **(5)** The time to perform the technique was adequate after the educational intervention.

## Introduction

Spread of micro-organisms through health professionals’ hands is one of the main factors related to Healthcare-Associated Infections (HAIs). This is why hand hygiene (HH) is an essential strategy to prevent these problems in places where care is provided to patients, in addition to being low-cost and effective. The World Health Organization (WHO) recommends five specific moments to reduce transmission risks, namely: before entering into contact with patients; before aseptic procedures; after exposure to body fluids; after entering into contact with patients; and after touching surfaces that are near patients^1^.

HAIs are adverse events acquired during or after performing procedures and/or hospitalizations; they impose a significant burden on health systems and raise care-related costs. These infections constitute one of the most frequent problems in the scope of health services and a substantial proportion of them results from the action of multidrug-resistant micro-organisms, imposing harms to patients, visitors and health professionals alike^2^.

It is estimated that HAIs account for nearly 3.5 million deaths a year and that they represent over a billion dollars to public coffers. The incidence of these adverse effects varies at the global level, reaching approximately 7% in developed countries, 8% in the European Union and up to 15% in underdeveloped nations. In this context, implementing interventions by means of multi-modal systems proves to be highly effective: it can avoid up to 821,000 deaths and provide significant economic returns, estimating that each dollar invested in HH promotion yields 24.6 dollars in benefits^3^.

Proper hand hygiene is a key measure not only in preventing HAI transmission but also in combating antimicrobial resistance, an increasing problem at the global level. Health professionals (especially Nursing ones) play an important role in bedside care management and in implementing health prevention measures. This involves following specific protocols established by the institutions and promoting adherence to the guidelines recommended by the WHO and by the National Health Surveillance Agency (*Agência Nacional de Vigilância Sanitária*, ANVISA), in addition to investing in ongoing education, ensuring that the entire team is updated about the best evidence-based practices^3^ ^-^ ^4^.

Consensus among authors points to low adherence to the HH practice among health professionals. A research study conducted in Brazil noticed that less than 50% of the nurses wash their hands properly and that the rate is even lower among nursing technicians, not exceeding 40%^5^ ^-^ ^6^. Similarly, a study that made 292 observations during a week revealed that adherence was only 37% among nursing assistants/technicians. These indicators can be considered a multi-factorial problem related to work overload, stress, inadequate physical environment or sinks and alcohol-based solution dispensers installed in wrong places, in addition to inadequate behavioral aspects^7^.

Ongoing health education has been widely used to improve adherence to the HH technique and its proper execution. However, challenges still persist, as the rates frequently return to their initial levels after traditional interventions. Innovating technologies such as interactive videos, Virtual Reality and gamification emerge as promising tools to promote sustainable changes in the professional practice^3^.

Gamification stands out because it incorporates game elements to the educational process and has shown potential to engage Nursing professionals, improve knowledge retention and enhance the execution of care-related practices^8^ ^-^ ^9^. However, the main gap this study seeks to bridge is the scarcity of research targeted at nurses and nursing technicians working directly in patient care, as most of the surveys on gamification are focused on Nursing students^8^ ^,^ ^10^ ^-^ ^12^. In addition to that, the existing research studies prioritize adherence to the five HH moments without considering technical quality of the execution, including proper use of alcohol-based solutions or liquid soap and hand massaging times, representing a limiting factor in assessing compliance with the guidelines in force^8^ ^,^ ^10^ ^-^ ^11^.

Given this scenario, the objective of the current study is to assess the effectiveness of a gamified educational intervention on proper execution of the Hand Hygiene technique among nurses and nursing technicians in hospitalization units.

## Method

### Type of study

This is a quasi-experimental study of the before-and-after type and with a non-randomized design^13^. The research was conducted using the same group both as Control and as Experimental, which implies stating that all the participants were subjected to both assessment conditions: the Pre-intervention and the Post-intervention periods.

### Study *locus*

The research was developed at a university hospital located in the *Zona da Mata Mineira* region, Brazil, which serves patients from the Unified Health System (*Sistema* Único *de Saúde*, SUS). The *locus* has 137 hospitalization beds (including 9 Intensive Care ones) and plays a fundamental role in providing healthcare to the local community, offering high-complexity services; in addition, it is a training center for future health professionals. Choice of this institution was strategic, as it provides broad interaction among teaching, research and assistance, with a focus on the quality of the care offered to the patients from the region.

### Period

The study was carried out from June to December 2024.

### Population

The population was comprised by nurses and nursing technicians from the Intensive Care for Adults, Medical Clinic and Surgical Clinic units, the exploratory focus of this study.

### Selection criteria

The inclusion criteria were as follows: nurses or nursing technicians with at least one year of experience in the profession. This criterion regarding effective professional performance time was considered important due to having sufficient practical experience to assess the HH technique and familiarity with the institutional protocols, thus conferring greater precision to the results. Professionals in administrative roles and residents were excluded from the study.

### Definition of the sample

Sample calculation was made in G*Power based on the t-test family, for bilateral comparisons between two groups specifically. The effect size was defined at 0.50, considered medium, with a 0.05 sampling error and 0.80 statistical power. These specifications were selected to ensure that the study had an adequate probability of detecting a significant difference (if there was any), minimizing the chances of Type II errors. After incorporating these parameters, the result indicated that a total sample size of 34 participants was necessary. This value was reached with a 0.8077 effective power, confirming that the sample calculated was suitable for the study requirements. However, in order to improve soundness of the results and compensate for possible sample losses, 45 Nursing professionals were included.

### Data collection

The data were collected by the researchers themselves through direct approaches in the research *locus* to invite nurses and nursing technicians to take part in the study. The process was divided into three phases, namely: Pre-intervention; Gamified educational intervention; and Post-intervention.

*Phase 1: Pre-intervention*

As a first step, the professionals were instructed to perform the HH technique according to their usual practice. In order to assess coverage and distribution of the technique used, an Optiglow^®^ solution was applied. This solution simulates the presence of micro-organisms and can be seen under fluorescent light. Fluorescence indicates proper product application, reflecting adequate execution of the technique on the right areas, either with the alcohol-based solution or with liquid soap. The participants were instructed to perform the technique as if they were using one of these products, without the need to actually apply them.

*Phase 2: Gamified educational intervention*

After the initial assessment, the educational intervention was implemented in a private environment, minimizing external influences. The training resorted to gamification as a pedagogical strategy to favor learning in a visual and interactive way. The participants were instructed individually or in pairs using the Hiji Sushi game, available on the *Educare* platform belonging to the Oswaldo Cruz Foundation (Fiocruz). The game simulates the movements recommended by the WHO for proper hand hygiene, presenting challenges where the professionals only progress to the next stage when properly complying with each step. The platform was accessed online by means of a tablet-type device (a portable piece of equipment with a touchscreen), allowing direct interaction with the digital content. In order to ensure homogeneity in using the tool, all the participants were previously trained in how to handle it, guaranteeing a uniform familiarity level with the technology^14^ ^-^ ^15^.

After the intervention, the Hiji Sushi game objective, the five HH moments recommended by the WHO and the importance of this practice in preventing HAIs were discussed with the participants^14^ ^-^ ^15^. Certain time was allowed to clear doubts. Subsequently, the professionals were dismissed so that they could resume their activities. A minimum interval of one hour was defined between the intervention and the reassessment, allowing the professionals to return to their usual activities. The objective of this period was to reduce the effect exerted by the hyperfocus generated by the educational activity, enabling the participants to resume their routines to subsequently being reassessed in a context that was closer to their everyday practice. Thus, the intention was to make a more faithful analysis regarding knowledge retention and about how the recommended movements were incorporated to the HH technique.

*Phase 3: Post-intervention*

After the interval, the participants returned to the private environment for a new HH technique assessment, using the same procedures as in the Pre-intervention phase

Developed based on the WHO recommendations^14^, the collection instrument was used to record diverse information about the participants and regarding execution of the HH technique. The variables assessed included the following: sociodemographic characteristics, fluorescent product coverage and distribution on the hands and number of stages forgotten while executing the technique, such as applying soap to the palms of the hands, massaging the interdigital spaces, rubbing the back of the fingers and massaging the fingertips and nails, among others. Execution of the technique was classified in a categorical way: Adequate, when all the stages were properly implemented; and Ineffective, when any stage was omitted or not properly implemented.

The time spent in the HH technique was initially recorded with a stopwatch and subsequently categorized into specific time ranges according to the recommendations for each type of technique. The time recommended for the alcohol-based solution is between 20 and 30 seconds; in turn, the suitable range for liquid soap is from 40 to 60 seconds^14^. Thus, times below the recommendations, over the maximum limit or between 30 and 39 seconds were not deemed adequate, as they fail to comply with the standards established for the technique to be effective. For the statistical analysis, time was treated as a continuous variable in the comparison between the Pre- and Post-intervention periods.

### Data treatment and analysis

The data collected were analyzed by means of different statistical tests. Descriptive statistics was initially employed to characterize the sociodemographic variables. Data normality was assessed using the Shapiro-Wilk test. In the absence of normal data distribution, it was decided to use Wilcoxon’s Signed-Rank test for the analysis. In addition to that, the effect size was also considered, interpreted according to the following criteria: values over 0.2 indicate a small effect, those over 0.5 point to a medium effect and those over 0.8 represent a large effect^16^. In order to assess changes in proper execution of the HH technique throughout the study, the McNemar analysis was applied, verifying alterations in the binary answers (Yes or No) before and after the intervention. All the statistical analyses were performed in the Statistical Package for the Social Sciences (SPSS) software, version 21.

### Ethical aspects

This study was approved by a Research Ethics Committee (*Comitê de* Ética *em Pesquisa*, CEP) under Opinion No. 6,600,099. All the participants signed a Free and Informed Consent Form (FICF).

## Results

The sample was comprised by 45 professionals: 31 (68.9%) nursing technicians and 14 (31.1%) nurses. As for gender, 31 (68.9%) were female and 14 (31.1%) were male. In relation to skin color, 21 (46.7%), 16 (35.6%) and 8 (17.8%) self-identified as brown-, white- and black-skinned, respectively. Referring to performance sectors, 19 (42.2%) worked in the Surgical Unit, 13 (28.9%) did so in the Intensive Care Unit and 13 (28.9%) were allocated to the Medical Clinic Unit. Regarding hand hygiene training, 35 (77.8%) reported having already took part in some session, whereas 10 (22.2%) had never undergone any training. Among those who had undergone training, 16 (35.6%) had done so more than 1 year ago, 14 (31.1%) less than 6 months ago and 6 (13.3%) between 6 months and 1 year ago; in addition to that, 9 (20%) of the professionals stated not having any previous training on the topic.

During the Pre-intervention period, the analysis revealed that the left hand palm was the area most frequently illuminated by the fluorescent light among the nursing technicians, with 19 records (61.3%), followed by the right hand palm with 16 (51.6%). The left and right hand fingertips were observed in 15 (48.4%) and 13 (41.9%) cases, respectively; in turn, the right and left thumbs were inspected in 12 (38.7%) and 11 (35.5%) records. Among the nurses, the back of the right hand was the area with the highest product concentration, with nine instances (64.3%), followed by the back of the left hand with seven (50,0%) and by the right hand palm with six cases (42.9%). In the fluorescent light simulator, the most illuminated areas indicate product concentration; in other words, regions that received more attention during HH. In turn, the least illuminated areas reflect neglected points. During the Post-intervention period, product distribution was uniform in both categories, indicating an improvement in the way the technique was performed. However, there were product concentration records in the right thumbs among the technicians, with 10 (32.3%) observations, followed by the right and left hand palms with nine (29.0%) and eight (25.8%) cases, respectively. Among the nurses, only four records (28.6%) were observed in the left thumbs.

In this context, the results indicate that the educational intervention exerted a positive impact in relation to reducing the number of areas with fluorescent product concentrated in specific sites, being lower in the Post-intervention stage (*M*=1.46; *SD*=1.15) when compared to the Pre-intervention (*M*=3.24; *SD*=1.62), with *z*=-5.450, *p*<0.001 and a large effect size (*r*=0.81).

The results showed a statistically significant change (*p*<0.001), indicating that more subjects followed the stages recommended by the WHO after the intervention. At the Pre-intervention moment, 25 (55.6%) of the Nursing professionals failed to follow all the recommended steps, namely: 19 (61.3%) technicians and 6 (42.9%) nurses. An expressive improvement was noticed after the gamified intervention: 44 (97.7%) of the professionals started to execute the technique properly, especially all 14 (100%) nurses, with only 1 (2.3%) nursing technician still not performing it correctly.

The number of stages forgotten while performing the HH technique was assessed before and after the educational intervention between the professional categories. The results showed that fewer stages were forgotten after the intervention (*M*=0.02; *SD*=0.14) when compared to the Pre-intervention period (*M*=0.80; *SD*=0.84), with *z*=-4.532, *p*<0.001 and a medium effect size (*r*=0.81) (Table 1).

**Table 1:** Stages forgotten during hand hygiene among Nursing professionals, before and after the educational intervention. Juiz de Fora, MG, Brazil, 2025

HH* stages, according to the WHO^†(^ ^15^	Description of the stages	Pre-intervention^‡^	Post-intervention
		n (%)	n (%)
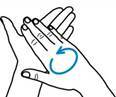	Apply soap to the palms of the hands, massaging them against one another	05 (12.8)	-
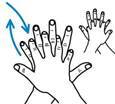	Rub the right hand palm against the left hand back interlacing the fingers, and vice versa	02 (5.1)	-
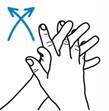	Interlace the fingers and massage the interdigital spaces	09 (33.0)	-
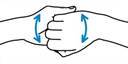	Rub the back of the fingers from one hand with the palm of the opposite hand, holding the fingers and with a rocking movement, and vice versa	01 (5.9)	-
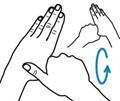	Rub the left thumb with the right hand palm and a circular movement, and vice versa	07 (27.9)	-
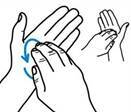	Massage the right hand fingertips and nails against the left hand palm with a circular movement, and vice versa	12 (37.2)	01 (2.2)
	Subjects not forgetting any stage	20 (77.9)	44 (97.8)

*HH = Hand Hygiene; ^†^WHO = World Health Organization; ^‡^More than one stage forgotten was observed per each Nursing professional evaluated

It is noted that the time to perform the HH technique was longer after the intervention among nurses and nursing technicians, following the guidelines proposed by the WHO (Table 2). The educational intervention proved to be effective, as the professionals executed the technique in the suitable time during the Post-intervention period (*M*=3.55; *SD*=0.50) when compared to the Pre-intervention stage (*M*=3.18; *SD*=0.80), with *z*=-2.441, *p*=0.015 and a small effect size (*r*=0.36).

**Table 2:** Assessment corresponding to the time to perform the hand hygiene technique among the Nursing professionals, as recommended by the WHO*, for the Pre- and Post-intervention periods (n = 45). Juiz de Fora, MG, Brazil, 2025

Time	Pre-intervention n (%)			Post-intervention n (%)	
	Nur. Tech.^†^	Nur.^‡^		Nur. Tech.^†^	Nur.^†^
Less than 20 seconds	01 (3.2)	0 (0)		-	-
Between 20 and 30 seconds^§^	07 (22.6)	01 (7.10)		11 (35.5)	9 (64.3)
Between 40 and 60 seconds^ǀǀ^	14 (45.2)	04 (28.6)		20 (64.5)	5 (35.7)
More than 60 seconds	09 (29.0)	09 (64.3)		-	-

*World Health Organization; ^†^Nursing Technicians; ^‡^Nurses; ^§^Suitable time recommended by the WHO for hand hygiene with alcohol-based solution; ^ǀǀ^Suitable time recommended by the WHO for hand hygiene with liquid soap

## Discussion

This study identified that the product was unevenly applied to the Nursing professionals’ hands before the gamified educational intervention and that it was more concentrated on the palms, fingertips and thumbs. Uniform distribution was verified after the intervention, with a significant reduction in the areas that presented higher application density at the first moment. These findings show that the educational intervention was effective in promoting more uniform and adequate product coverage. In addition to that, more stages were properly followed and fewer ones were forgotten while performing the HH technique. The time to execute the technique was also longer, in line with the WHO recommendations and highlighting the effectiveness of the intervention aided by gamification.

Gamification has been increasingly applied in ongoing health education, exerting positive impacts on learning among professionals from different areas. In addition to favoring the acquisition of cognitive, procedural and behavioral skills, gamified strategies in clinical and in-hospital contexts result in increased engagement, information retention and adherence to protocols. The number of materials published on gamification in health has increased significantly, evidencing the topic as emerging and with a focus on central concepts such as game-based learning and e-learning. Consequently, gamification represents an expanding approach capable of continuously supporting professional development and the acquisition of essential skills for a safe clinical practice^17^ ^-^ ^18^.

Developed by Fiocruz, the Hiji Sushi game was used as a strategy for HH teaching in this study, showing a positive initial impact on learning. In this sense, gamification has proved to be an innovating health education tool, promoting cognitive development, acquisition of information and behavioral changes. A study points out the significant benefits contributed by gamified interventions in the teaching-learning process; however, no other surveys were identified in the literature using this game specifically to teach Hand Hygiene, underscoring novelty of the approach^18^.

The current study corroborates findings on gamification as an effective strategy in HH teaching, assessing the quality of the technique executed, its execution time, the stages forgotten and the areas most frequently illuminated by the fluorescent light simulator. Although not targeted at HAI prevention and control, a study conducted with the “Oncologic Emergencies” game showed a positive impact when using the gamified approach in simulating cancer emergencies. The assessment by means of pre- and post-tests indicated a significant improvement in the participants’ performance, revealing that, in addition to consolidating knowledge and improving clinical reasoning, gamification enhances interest, motivation and satisfaction in learning^19^.

From another perspective, a study investigated a game targeted at developing diagnostic skills in Nursing with the objective of providing a controlled environment where the students could practice clinical analysis and decision-making, simulating real-world work situations. The results indicated that the participants who used the game were more confident in real-world clinical scenarios, evidencing the relevance of gamification in the professionals’ ongoing education^20^. It is worth noting that, although there is abundant literature on gamified educational interventions in various knowledge areas and although hand hygiene is a simple and effective technique for HAI prevention and control, it can become more complex when involving individual aspects, as well as those related to the work environment, the teams, the tasks, the patients, organization and management^21^.

It was possible to notice that, when taking part in the game, the nurses and nursing technicians reflected about the recommended areas they had forgotten in the Pre-intervention stage. The WHO recommends a six-step HH technique (either with an alcohol-based solution or with liquid soap) to ensure microbial load reductions; in turn, a systematic review confirmed its efficacy but detected heterogeneity across the studies and a high risk of bias, indicating the need for more research studies to determine the most effective and viable technique^22^.

In this sense, when accessing the game, the participants find themselves in an environment with all the items required for HH and can only progress in the phases if they follow the six stages recommended by the WHO^14^ ^,^ ^22^. These aspects are in line with the literature, which indicates that, in addition to having gained notoriety in the last few years, using games in Nursing education also highlighted recent trends and future prospects to use gamified interventions in the teaching-learning process^20^.

The fluorescent simulator showed insufficient product distribution on the hands, with more concentration on the palms and thumbs. Its application was more balanced and well-distributed after the intervention. A pilot study used thermal images to assess HH with a focus on solution distribution uniformity, reflecting coverage of the areas cleaned. The images were effective in identifying neglected areas such as interdigital spaces, fingertips and thumbs, frequently forgotten and with high cross-contamination risks. This research revealed that even experienced professionals made mistakes; in addition, the authors highlight the potential of technologies in training and monitoring hand hygiene practices^23^. For this reason, the recommendation is to incorporate these technologies as an educational strategy to reinforce awareness and refine the Nursing professionals’ technique.

The gamified educational intervention described in this research resulted in a significant improvement in proper execution of the HH technique. When combined with digital technologies, multi-modal strategies expanded learning accessibility and flexibility. Playful and interactive methods proved to exert a positive impact, promoting behavioral changes and favoring the HH practice, especially when adapted to the professional context^24^. A quasi-experimental study that assessed the effectiveness of an educational intervention on the same topic in a hospital X-ray unit included lectures, visual materials and alcohol gel dispensers. After the intervention there was an increase from 28.9% to 51.4% in terms of knowledge, acceptance of the product used and perception of the need to execute the technique after entering into contact with areas near the patients^25^.

Although knowledge among Nursing professionals is from moderate to good, the need is underscored for ongoing educational programs that promote exchanges of experiences among team members^26^. In an Intensive Care Unit (ICU) from Cuiabá, Brazil, the HH technique was more frequently executed after providing assistance; in turn, such frequency was lower before entering into contact with the patients and glove use exerted a negative impact on promoting the proper technique^24^. The findings detected in this research indicate that implementing gamified educational interventions can improve quality of the technique, especially at the five critical moments recommended by the WHO, in addition to contributing to patient safety in the clinical practice^14^.

The analysis revealed an impact on the time nurses and nursing technicians spend on HH. According to the WHO recommendations, alcohol-based solutions should be used for 20-30 seconds, whereas hygiene procedures with liquid soap and water should last 40-60 seconds. Although there may be differences in the application context, it is important to note that alcohol gel is recommended with lower soil levels, whereas soap and water are indicated for visible dirt situations^14^.

In this study, the HH execution time was compliant with the recommendations in force (varying from 20 to 60 seconds), according to the simulation of using an alcohol-based solution or liquid soap and considering the specifications of the products employed. In the Post-intervention assessment, all the participants remained within the recommended time (20-30 seconds for the alcohol-based solution and 40-60 seconds for liquid soap) showing better alignment with the international guidelines^2^ ^,^ ^14^ ^,^ ^27^. Proper execution of the technique within the recommended time is fundamental to ensure full coverage of all hand surfaces, reinforcing the potential of educational interventions in promoting proper compliance with this practice^14^ ^,^ ^28^.

This research presents some limitations, such as the fact that it was conducted in a single hospital, which restricts generalization of the findings. In addition to that, the quasi-experimental design of the before-and-after type with a brief interval of only one hour between assessments precludes evaluating the medium- and long-term impact exerted by the intervention, influencing understanding about knowledge retention and sustainability of the behavioral changes. Not having implemented a longitudinal follow-up also precludes analyzing if the effects observed were maintained over time. The direct observation of the participants may have been influenced by the Hawthorne effect; however, this risk was minimized by means of standardized routines, allowing the professionals to resume their usual activities before the reassessment.

The contributions this study makes to the clinical practice reinforce the importance of incorporating innovating educational strategies such as gamification to improve proper execution of the HH technique among nurses and nursing technicians. Implementing playful and interactive approaches can ease learning, rendering training more appealing and effective, in addition to promoting sustainable behavioral changes. Adopting technologies such as fluorescent simulators and digital games in health education contributes to raising awareness about team members following the proper technique. These findings underscore the need to integrate active methodologies into ongoing education programs, strengthening evidence-based practices and qualifying the care provided to the patients.

## Conclusion

It was concluded that the gamified educational intervention was effective in proper execution of the hand hygiene technique among nurses and nursing technicians in hospitalization units. The following stood out among the effects observed: more uniform fluorescent product distribution on the hands; fewer stages forgotten; and improved quality in executing the technique, including compliance with the times recommended by the World Health Organization, both with the alcohol-based solution and with liquid soap. These findings evidence the effectiveness of game-based health education in promoting safer and more effective practices, showing potential to contribute in reducing the risk of healthcare-associated infections. It is noted that the findings are of a baseline nature and that studies with larger samples, in different contexts and with long-term monitoring are required.

## Data Availability

All data generated or analysed during this study are included in this published article.

## References

[1] Chen J, Yang L, Mak YW, O’Donoghue M, Shi C, Tsang H (2024). Hand hygiene education components among first-year nursing students: a cluster randomized clinical trial. JAMA Netw Open.

[2] Classen DC, Rhee C, Dantes RB, Benin AL (2024). Healthcare-associated infections and conditions in the era of digital measurement. Infect Control Hosp Epidemiol.

[3] World Health Organization (2025). The case for investment and action in infection prevention and control.

[4] Fernandes DR, Santos BN, Guimarães CS, Ferreira EB, Margatho AS, Reis PEDD (2024). Educational technologies for teaching hand hygiene: systematic review. PLoS One.

[5] Afework A, Tamene A (2025). Uncovering the obstacles: a comprehensive analysis of barriers to hand hygiene adherence among healthcare providers: a systematic review. BMC Infect Dis.

[6] Alvim ALS, Reis LC, Couto BRGM, Starling CEF, Vaz R (2019). Evaluation of hand hygiene practices in three intensive care units. Rev Epidemiol Control Infect.

[7] Valim MD, Rossetto JR, Bortolini J, Herwaldt L (2024). Hand hygiene compliance in a Brazilian COVID-19 unit: the impact of moments and contact precautions. Antimicrob Resist Infect Control.

[8] Sanz-Martos S Álvarez-García C, Álvarez-Nieto C López-Medina IM, López-Franco MD Fernandez-Martinez ME (2024). Effectiveness of gamification in nursing degree education. PeerJ.

[9] Bass GA, Chang CWJ, Sorce LR, Subramanian S, Laytin AD, Somodi R (2024). Gamification in critical care education and practice. Crit Care Explor.

[10] Rushdan EE, Mohamed MAE, Abdelhalim GE, El-Ashry AM, Ali HFM (2025). Effect of an escape room as a gamification evaluation tool on clinical reasoning and teamwork skills among nursing students: a quasi-experimental study. Nurse Educ Pract.

[11] Delage PEGA, Mendes ES, Paula JGF, Mendes ISB, Almeida MS, Costa FNA (2021). Creation and application of a gamified strategy in nursing undergraduate education. Cogitare Enferm.

[12] Castro TC, Gonçalves LS (2018). The use of gamification to teach in the nursing field. Rev Bras Enferm.

[13] Polit DF, Beck CT (2019). Fundamentos da pesquisa em enfermagem: avaliação de evidências para a prática de enfermagem.

[14] World Health Organization (2009). Guide to implementation: a guide to the implementation of the WHO multimodal hand hygiene improvement strategy.

[15] Valle TZ, Magalhães TS, Ribeiro RA, Sousa GLSC, Furtado BL (2019). Hiji Sushi.

[16] Kallogjeri D, Piccirillo JF (2023). A simple guide to effect size measures. JAMA Otolaryngol Head Neck Surg.

[17] Davis K, Gowda AS, Thompson-Newell N, Maloney C, Fayyaz J, Chang T (2024). Gamification, serious games, and simulation in health professions education. Pediatr Ann.

[18] Silva LFM, Granito CCD (2024). A aplicação da gamificação como ferramenta pedagógica na formação de enfermeiras e enfermeiros. Journal of Media Critiques.

[19] Dönmez AA, Çalik A, Terzi K, Kapucu S (2025). Designing and evaluating ONCologic EMergencies escape room game for undergraduate nursing students: the ONCEM quasi-experimental pilot study. Educ Inf Technol.

[20] Tinôco JDS, Silva LSD, Medeiros TM, Grande MEG, Guedes MLA, Fernandes MICD (2023). “Enfermeiro Diagnosticador” board game for teaching diagnostic reasoning in nursing: a quasi-experimental study. Acta Paul Enferm.

[21] Alshagrawi S, Alhodaithy N (2024). Determinants of hand hygiene compliance among healthcare workers in intensive care units: a qualitative study. BMC Public Health.

[22] Price L, Gozdzielewska L, Matuluko A, Pittet D, Allegranzi B, Reilly J (2022). Comparing the effectiveness of hand hygiene techniques in reducing the microbial load and covering hand surfaces in healthcare workers: updated systematic review. Am J Infect Control.

[23] Boyce JM, Martinello RA (2022). Pilot study of using thermal imaging to assess hand hygiene technique. Am J Infect Control.

[24] Valim MD, Reis GF, Santos BS, Goulart LS, Bortolini J, Cardoso JDC (2024). Adherence to hand hygiene technique: an observational study. Acta Paul Enferm.

[25] O’Donoghue M, Ng SH, Suen LK, Boost M (2016). A quasi-experimental study to determine the effects of a multifaceted educational intervention on hand hygiene compliance in a radiography unit. Antimicrob Resist Infect Control.

[26] Blomgren PO, Leo Swenne C, Lytsy B, Hjelm K (2024). Hand hygiene knowledge among nurses and nursing students: a descriptive cross-sectional comparative survey using the WHO’s “Hand Hygiene Knowledge Questionnaire”. Infect Prev Pract.

[27] Tartari E Bellissimo-Rodrigues F, Pires D Fankhauser C, Lotfinejad N Saito H (2024). Updates and future directions regarding hand hygiene in the healthcare setting: insights from the 3rd ICPIC alcohol-based handrub (ABHR) task force. Antimicrob Resist Infect Control.

[28] Alvim ALS (2024). Hand hygiene in health services: reflection on history and concepts. Enferm Foco.

