# Supplementary material for: Effectiveness of a gamified educational intervention on hand hygiene among Nursing professionals: a quasi-experimental study
Source: Rev Lat Am Enfermagem. 2026 Aug 10;34:e4891. doi: 10.1590/1518-8345.8067.4891 (PMC13459839; doi:10.1590/1518-8345.8067.4891)
Supplement: Translation

# Translation: Efetividade de intervenção educativa gamificada na higienização das mãos entre profissionais de enfermagem: estudo quase-experimental

**DOI:** 10.1590/1518-8345.8067.4892

**Authors:** Maiara Luci Silva Costa, Márcio Medeiros, Fábio da Costa Carbogim, Thiago César Nascimento, André Luiz Silva Alvim

**Affiliations:** 1 Universidade Federal de Juiz de Fora, Juiz de Fora, MG, Brasil.; 2 Universidade Federal de Juiz de Fora, Faculdade de Enfermagem, Juiz de Fora, MG, Brasil.; 3 Universidade Federal de Juiz de Fora, Faculdade de Enfermagem, Departamento de Enfermagem Aplicada, Juiz de Fora, MG, Brasil.; 4 Universidade Federal de Juiz de Fora, Faculdade de Enfermagem, Departamento de Enfermagem Básica, Juiz de Fora, MG, Brasil.

**Keywords:** Desinfecção das Mãos, Equipe de Enfermagem, Gamificação, Educação em Saúde, Educação, Controle de Infecções

## Abstract

**Objetivo:**

avaliar a efetividade de uma intervenção educativa gamificada na execução correta da técnica de higienização das mãos entre enfermeiros e técnicos de enfermagem em unidades de internação hospitalar.

**Método:**

trata-se de um estudo quase-experimental, do tipo antes e depois, conduzido com 45 profissionais de enfermagem. A coleta de dados foi realizada por meio de um formulário estruturado para a observação da técnica nos períodos pré e pós-intervenção. A análise estatística dos dados foi realizada utilizando o teste de Wilcoxon Signed-Rank e o teste de McNemar.

**Resultados:**

no período pré-intervenção, 61,3% dos técnicos e 42,9% dos enfermeiros não seguiam todos os passos da técnica; após a gamificação, 100% dos enfermeiros e 97,7% dos técnicos de enfermagem realizaram os passos corretos (*p*<0,001), com redução no número de etapas esquecidas (*z*= 4,532, *p*<0,001, *r*=0,81). O tempo de execução também foi adequado após a intervenção, seguindo as recomendações da Organização Mundial da Saúde (*z*=-2,441, *p*=0,015, *r*=0,36).

**Conclusão:**

a intervenção educativa gamificada foi efetiva na execução correta da técnica de higienização das mãos entre enfermeiros e técnicos de enfermagem, reduzindo erros e aumentando a conformidade com as diretrizes internacionais.

## Destaques

**(1)** A gamificação aumentou a adesão à higienização das mãos realizada pela enfermagem. **(2)** A gamificação demonstrou impacto positivo na aprendizagem e adesão à técnica. **(3)** Houve redução significativa das etapas esquecidas durante a higienização das mãos. **(4)** A distribuição do produto nas mãos tornou-se mais uniforme após a intervenção. **(5)** O tempo de execução da técnica foi adequado após a intervenção educativa.

## Introdução

A transmissão de microrganismos pelas mãos dos profissionais de saúde é um dos principais fatores associados às Infecções Relacionadas à Assistência à Saúde (IRAS). Por esse motivo, a higienização das mãos (HM) é uma estratégia essencial para prevenir esses agravos nos locais de assistência ao paciente, sendo simples, de baixo custo e eficaz. A Organização Mundial da Saúde (OMS) recomenda cinco momentos específicos para reduzir o risco de transmissão, sendo eles: antes do contato com o paciente, antes de procedimentos assépticos, após exposição a fluidos corporais, após o contato com o paciente e após o toque em superfícies próximas a ele^1^.

As IRAS são eventos adversos adquiridos durante ou subsequente à realização de procedimentos e/ou hospitalizações e promovem uma carga significativa aos sistemas de saúde e elevam os custos assistenciais. Essas infecções constituem um dos problemas mais frequentes no âmbito dos serviços de saúde, com uma parcela substancial delas resultante da ação de microrganismos multirresistentes, acarretando prejuízos tanto a pacientes quanto a visitantes e profissionais de saúde^2^.

Estima-se que as IRAS sejam responsáveis por cerca de 3,5 milhões de mortes anualmente e apresentem custos superiores a um bilhão de dólares aos cofres públicos. A incidência desses eventos adversos varia globalmente, atingindo aproximadamente 7% em países desenvolvidos, 8% na União Europeia e chegando a 15% em países em desenvolvimento. Nesse contexto, a implementação de intervenções por meio de sistemas multimodais revela-se altamente eficaz, podendo evitar até 821 mil óbitos e proporcionar um retorno econômico significativo, estimando-se que a cada 1 dólar investido na promoção da HM gere um ganho de 24,6 dólares^3^.

A higienização adequada das mãos é uma medida essencial não apenas na prevenção da transmissão de IRAS, mas também no combate à resistência antimicrobiana, um problema crescente mundialmente. Os profissionais de saúde, especialmente da enfermagem, têm um papel importante na gestão da assistência à beira-leito e na implementação de medidas de prevenção em saúde. Isso envolve seguir os protocolos específicos estabelecidos pelas instituições, promover a adesão às diretrizes recomendadas pela OMS e pela Agência Nacional de Vigilância Sanitária (Anvisa), além de investir em educação continuada, garantindo que toda a equipe esteja atualizada sobre as melhores práticas baseadas em evidências^3^ ^-^ ^4^.

Um consenso entre autores aponta uma baixa adesão dos profissionais de saúde à prática de HM. Pesquisas realizadas no Brasil observaram que menos de 50% dos enfermeiros realizam a higiene das mãos adequadamente, e entre os técnicos de enfermagem a taxa é ainda mais baixa, não ultrapassando 40%^5^ ^-^ ^6^. De maneira similar, um estudo com 292 observações durante uma semana revelou que a adesão dos auxiliares/técnicos de enfermagem foi de apenas 37%. Esses indicadores podem ser considerados um problema multifatorial, relacionado à sobrecarga de trabalho, estresse, ambiente físico inadequado, má localização das pias e dispensadores de preparação alcoólica, além de fatores comportamentais inadequados^7^.

A educação permanente em saúde tem sido amplamente utilizada para aumentar a adesão e a execução adequada da técnica de HM. Contudo, os desafios persistem, pois os índices frequentemente retornam aos níveis iniciais após intervenções tradicionais. Tecnologias inovadoras, como vídeos interativos, realidade virtual e gamificação, emergem como ferramentas promissoras para promover mudanças sustentáveis na prática profissional^3^.

A gamificação destaca-se ao incorporar elementos de jogos no processo educativo e tem demonstrado potencial para engajar profissionais de enfermagem, aprimorar a retenção do conhecimento e melhorar a execução de práticas assistenciais^8^ ^-^ ^9^. No entanto, a principal lacuna que este estudo busca preencher é a escassez de pesquisas voltadas para enfermeiros e técnicos de enfermagem que atuam diretamente na assistência ao paciente, enquanto a maior parte das pesquisas sobre gamificação concentra-se em estudantes de enfermagem^8^ ^,^ ^10^ ^-^ ^12^. Além disso, os estudos existentes priorizam a adesão aos cinco momentos da HM, sem considerar a qualidade técnica da execução, incluindo o uso adequado de preparações alcoólicas ou sabonete líquido e o tempo de fricção das mãos, sendo um fator limitante na avaliação da conformidade com as diretrizes vigentes^8^ ^,^ ^10^ ^-^ ^11^.

Diante desse cenário, este estudo tem como objetivo avaliar a efetividade de uma intervenção educativa gamificada na execução correta da técnica de higienização das mãos entre enfermeiros e técnicos de enfermagem em unidades de internação hospitalar.

## Método

### Tipo de estudo

Trata-se de um estudo quase-experimental, do tipo antes e depois, com um desenho não randomizado^13^. A pesquisa foi conduzida utilizando o mesmo grupo tanto como controle quanto experimental, o que implica dizer que todos os participantes passaram pelas duas condições de avaliação: o período pré-intervenção e o período pós-intervenção. 

### Local de estudo

A pesquisa foi realizada em um hospital universitário localizado na região da Zona da Mata Mineira, Brasil, que atende a pacientes do Sistema Único de Saúde (SUS). O local possui 137 leitos de internação, incluindo nove de Terapia Intensiva, e desempenha papel fundamental no atendimento à saúde da comunidade local, oferecendo serviços de alta complexidade, além de ser um centro de formação para futuros profissionais de saúde. A escolha dessa instituição foi estratégica, pois proporciona ampla interação entre ensino, pesquisa e assistência, com foco na qualidade do atendimento aos pacientes da região.

### Período

O estudo foi realizado no período de junho a dezembro de 2024.

### População

A população foi constituída por enfermeiros e técnicos de enfermagem das Unidades de Terapia Intensiva Adulto, Clínica Médica e Clínica Cirúrgica, sendo o foco exploratório deste estudo. 

### Critérios de seleção

Os critérios de inclusão foram: ser enfermeiro ou técnico de enfermagem com, no mínimo, um ano de experiência na profissão. Esse critério de tempo de efetivo exercício profissional foi considerado importante devido à vivência prática suficiente para a avaliação da técnica de HM e familiaridade com os protocolos institucionais, gerando, assim, maior precisão nos resultados. Profissionais com funções administrativas ou residentes foram excluídos do estudo.

### Definição da amostra

O cálculo amostral foi realizado no *software* G*Power, com base na família de teste t, especificamente para comparações bilaterais entre dois grupos. Optou-se por um tamanho de efeito de 0,50, considerado médio, com um erro amostral de 0,05 e um poder estatístico de 0,80. Essas especificações foram selecionadas para garantir que o estudo tivesse uma probabilidade adequada de detectar uma diferença significativa, caso ela existisse, minimizando a possibilidade de erro tipo II. Após a inserção desses parâmetros, o resultado indicou a necessidade de um tamanho total de amostra de 34 participantes. Esse valor foi alcançado com um poder efetivo de 0,8077, confirmando a adequação da amostra calculada às necessidades do estudo. No entanto, para aumentar a robustez dos resultados e compensar possíveis perdas amostrais, foram incluídos 45 profissionais de enfermagem.

### Coleta de dados

A coleta de dados foi realizada pelos próprios pesquisadores, por meio de abordagens diretas no local de pesquisa para convidar enfermeiros e técnicos de enfermagem a participarem do estudo. O processo foi dividido em três fases: pré-intervenção, intervenção educativa gamificada e pós-intervenção.

*Fase 1: Pré-intervenção*

Inicialmente, os profissionais foram orientados a realizar a HM conforme sua prática habitual. Para avaliar a cobertura e a distribuição da técnica utilizada, foi aplicada a solução Optiglow^®^, que simula a presença de microrganismos e pode ser visualizada sob luz fluorescente. A fluorescência indica a aplicação correta do produto, refletindo a execução adequada da técnica, seja com preparação alcoólica ou com sabonete líquido, nas áreas apropriadas. Os participantes foram instruídos a executar a técnica como se estivessem utilizando um desses produtos, sem a necessidade de aplicação real.

*Fase 2: Intervenção educativa gamificada*

Após a avaliação inicial, a intervenção educativa foi conduzida em um ambiente reservado, minimizando influências externas. O treinamento utilizou gamificação como estratégia pedagógica para favorecer a aprendizagem de maneira visual e interativa. Os participantes foram instruídos individualmente ou em duplas, utilizando o jogo Hiji Sushi, disponível na plataforma Educare da Fundação Oswaldo Cruz (Fiocruz). O jogo simula os movimentos preconizados pela OMS para a higiene adequada das mãos, apresentando desafios em que os profissionais só avançam para a próxima etapa ao realizar corretamente cada passo. O acesso à plataforma foi realizado de forma virtual, por meio de um dispositivo eletrônico do tipo tablet, equipamento portátil com tela sensível ao toque, permitindo a interação direta com o conteúdo digital. Para garantir homogeneidade no uso da ferramenta, todos os participantes foram previamente treinados quanto ao seu manuseio, assegurando um nível equitativo de familiaridade com a tecnologia^14^ ^-^ ^15^.

Após a intervenção, foi conduzida uma discussão com os participantes sobre o objetivo do jogo Hiji Sushi, os cinco momentos recomendados pela OMS para a HM e a importância dessa prática na prevenção de IRAS^14^ ^-^ ^15^. Um espaço foi aberto para esclarecimento de dúvidas. Foi estabelecido um intervalo mínimo de uma hora entre a intervenção e a reavaliação, permitindo que os profissionais retornassem às suas atividades habituais. Esse período teve como objetivo reduzir o efeito do hiperfoco gerado pela atividade educativa, possibilitando que os participantes retomassem sua rotina e, posteriormente, fossem reavaliados em um contexto mais próximo à sua prática cotidiana. Dessa forma, buscou-se uma análise mais fidedigna da retenção do conhecimento e da incorporação dos movimentos preconizados na HM.

*Fase 3: Pós-intervenção*

Após o intervalo, os participantes retornaram ao ambiente reservado para uma nova avaliação da técnica de HM, utilizando os mesmos procedimentos da fase pré-intervenção.

O instrumento de coleta, desenvolvido com base nas recomendações da OMS^14^, foi utilizado para registrar informações sobre os participantes e a execução da técnica de HM. Foram avaliadas variáveis como características sociodemográficas, cobertura e distribuição do produto fluorescente nas mãos e o número de etapas esquecidas durante a técnica, incluindo ensaboar as palmas, friccionar os espaços interdigitais, esfregar o dorso dos dedos, friccionar as polpas digitais e unhas, entre outras. A execução da técnica foi classificada de forma categórica: adequada, quando todas as etapas foram realizadas corretamente, e ineficaz, quando ocorreu falha ou omissão em qualquer etapa.

O tempo de realização da HM foi inicialmente registrado com um cronômetro e, posteriormente, categorizado em faixas temporais específicas de acordo com as recomendações para cada tipo de técnica. Para a preparação alcoólica, o tempo recomendado é entre 20 e 30 segundos, enquanto para o sabonete líquido, a faixa adequada é de 40 a 60 segundos^14^. Assim, tempos inferiores ao recomendado, superiores ao limite máximo ou entre 30 e 39 segundos não foram considerados adequados, pois não atendem aos padrões estabelecidos para a efetividade da técnica. Para análise estatística, o tempo foi tratado como variável contínua na comparação entre os períodos pré e pós-intervenção.

### Tratamento e análise dos dados

Os dados coletados foram analisados por meio de diferentes testes estatísticos. Inicialmente, foi empregada a estatística descritiva para caracterizar as variáveis sociodemográficas. A normalidade dos dados foi avaliada utilizando o teste de Shapiro-Wilk. Diante da ausência de distribuição normal dos dados, optou-se pelo uso do teste de Wilcoxon *Signed-Rank* para as análises. Além disso, foi considerado o tamanho de efeito, interpretado de acordo com os seguintes critérios: valores superiores a 0,2 indicam um efeito baixo, superiores a 0,5 indicam um efeito médio e superiores a 0,8 indicam um efeito alto^16^. Para avaliar mudanças na realização correta da técnica de HM ao longo do estudo, foi aplicada a análise de McNemar, que verificou as alterações nas respostas binárias (sim ou não) antes e após a intervenção. Todas as análises estatísticas foram realizadas utilizando o *software Statistical Package for the Social Sciences* (SPSS), versão 21.

### Aspectos éticos

Este estudo foi aprovado pelo Comitê de Ética em Pesquisa (CEP), sob número de parecer: 6.600.099. Todos os participantes leram e assinaram o Termo de Consentimento Livre e Esclarecido (TCLE).

## Resultados

A amostra foi composta por 45 profissionais, sendo 31 (68,9%) técnicos de enfermagem e 14 (31,1%) enfermeiros. Quanto ao sexo, 31 (68,9%) eram do sexo feminino e 14 (31,1%) do sexo masculino. Em relação à cor, 21 (46,7%) se identificaram como pardos, 16 (35,6%) como brancos e oito (17,8%) como pretos. No que diz respeito ao setor de atuação, 19 (42,2%) trabalhavam na Unidade Cirúrgica, 13 (28,9%) na Unidade de Terapia Intensiva e 13 (28,9%) na Unidade de Clínica Médica. Sobre o treinamento em higiene de mãos, 35 (77,8%) relataram já ter participado, enquanto 10 (22,2%) nunca realizaram nenhum. Entre os que receberam treinamento, 16 (35,6%) o realizaram há mais de um ano, 14 (31,1%) há menos de seis meses e seis (13,3%) entre seis meses e um ano; além disso, nove (20%) dos profissionais indicaram não possuir formação prévia sobre o tema.

Durante o período pré-intervenção, a análise revelou que, entre os técnicos de enfermagem, a palma da mão esquerda foi o local mais frequentemente iluminado pela solução fluorescente, com 19 registros (61,3%), seguida pela palma da mão direita, com 16 (51,6%). As polpas digitais da mão esquerda foram observadas em 15 casos (48,4%), as da mão direita em 13 (41,9%), o polegar da mão direita em 12 registros (38,7%) e da esquerda em 11 (35,5%). Entre os enfermeiros, o dorso da mão direita foi a região em que o produto mais se concentrou, com nove ocorrências (64,3%), seguido pelo dorso da mão esquerda, com sete (50,0%), e pela palma da mão direita, com seis casos (42,9%). No simulador fluorescente, áreas mais iluminadas indicam concentração do produto, ou seja, regiões que receberam maior atenção durante a HM, enquanto áreas menos iluminadas refletem pontos negligenciados. No período pós-intervenção, a distribuição do produto foi uniforme em ambas as categorias, indicando melhora na execução da técnica. Contudo, ainda houve registros de concentração do produto entre os técnicos no polegar da mão direita, com 10 (32,3%) observações, seguido pela palma da mão direita, com nove (29,0%) e da mão esquerda, com oito casos (25,8%). Entre os enfermeiros, apenas quatro registros (28,6%) foram observados no polegar da mão esquerda.

Nesse contexto, os resultados indicam que a intervenção educativa teve impacto positivo em relação à diminuição do número de áreas concentradas com o produto fluorescente em locais específicos, sendo menor na etapa pós-intervenção (*M*=1,46; *DP*=1,15) quando comparada à pré-intervenção (*M*=3,24; *DP*=1,62), com*z*=-5,450, *p*<0,001, e um tamanho de efeito alto (*r*=0,81).

Os resultados demonstraram uma mudança estatisticamente significativa (*p*<0,001), indicando que o número de pessoas que seguiram as etapas de acordo com a OMS aumentou após a intervenção. No momento pré-intervenção, 25 (55,6%) dos profissionais de enfermagem não seguiram todos os passos recomendados, sendo 19 (61,3%) técnicos e seis (42,9%) enfermeiros. Após a intervenção gamificada, observou-se uma melhora expressiva: 44 (97,7%) dos profissionais passaram a executar a técnica corretamente, em especial os 14 (100%) enfermeiros, restando apenas um (2,3%) técnico de enfermagem que ainda não a realizava de forma adequada. 

O número de etapas esquecidas durante a HM foi avaliado antes e após a intervenção educativa entre as categorias profissionais. Os resultados demonstraram que houve um menor número de etapas esquecidas pós-intervenção (*M*=0,02; *DP*=0,14) quando comparado ao período pré-intervenção (*M*=0,80; *DP*=0,84), com*z*=-4,532, *p*<0,001, e um tamanho de efeito médio (*r*=0,81) (Tabela 1).

**Tabela 1:** Etapas esquecidas durante a higienização das mãos pelos profissionais de enfermagem, pré e pós-intervenção educativa. Juiz de Fora, MG, Brasil, 2025

Etapas para HM*, de acordo com a OMS^†(^ ^15^	Descrição das etapas	Pré-intervenção^‡^	Pós-intervenção
		n (%)	n (%)
	Ensaboar as palmas das mãos, friccionando-as entre si	05 (12,8)	-
	Esfregar a palma da mão direita contra o dorso da mão esquerda entrelaçando os dedos e vice-versa	02 (5,1)	-
	Entrelaçar os dedos e friccionar os espaços interdigitais	09 (33,0)	-
	Esfregar o dorso dos dedos de uma mão com a palma da mão oposta, segurando os dedos, com movimento de vai e vem e vice-versa	01 (5,9)	-
	Esfregar o polegar esquerdo com o auxílio da palma da mão direita, fazendo movimento circular e vice-versa	07 (27,9)	-
	Friccionar as polpas digitais e unhas da mão direita contra a palma da mão esquerda, fazendo movimento circular e vice-versa	12 (37,2)	01 (2,2)
	Não esqueceu etapas	20 (77,9)	44 (97,8)

*HM = Higienização das mãos; ^†^OMS = Organização Mundial da Saúde; ^‡^Observou-se mais de uma etapa esquecida por profissional de enfermagem avaliado

Destaca-se que houve um aumento no tempo de HM pós-intervenção entre enfermeiros e técnicos de enfermagem, seguindo as diretrizes propostas pela OMS (Tabela 2). A intervenção educativa mostrou-se eficaz, com os profissionais realizando a técnica no tempo adequado no período pós-intervenção (*M*=3,55; *DP*=0,50), em comparação à etapa pré-intervenção (*M*=3,18; *DP*=0,80), com *z*=-2,441, *p*=0,015, e um tamanho de efeito baixo (*r*=0,36).

**Tabela 2:** Avaliação do tempo de higienização das mãos pelos profissionais de enfermagem, conforme preconizado pela OMS*, para os períodos pré e pós-intervenção (n = 45). Juiz de Fora, MG, Brasil, 2025

Tempo	Pré-intervenção n (%)			Pós-intervenção n (%)	
	Téc. Enf.^†^	Enf.^‡^		Téc. Enf.^†^	Enf.^‡^
Menos de 20 segundos	01 (3,2)	0 (0)		-	-
Entre 20 e 30 segundos^§^	07 (22,6)	01 (7,10)		11 (35,5)	9 (64,3)
Entre 40 e 60 segundos^ǀǀ^	14 (45,2)	04 (28,6)		20 (64,5)	5 (35,7)
Acima de 60 segundos	09 (29,0)	09 (64,3)		-	-

*Organização Mundial da Saúde; ^†^Técnicos de Enfermagem; ^‡^Enfermeiros; ^§^Tempo adequado preconizado pela OMS para higienização das mãos com preparação alcoólica; ^ǀǀ^Tempo adequado preconizado pela OMS para higienização das mãos com com sabonete líquido

## Discussão

Este estudo identificou que, antes da intervenção educativa gamificada, a aplicação do produto nas mãos dos profissionais de enfermagem era irregular, com maior concentração nas regiões palmares, polpas digitais e polegares. Após a intervenção, verificou-se uma distribuição uniforme, com redução significativa nas áreas que inicialmente apresentavam maior densidade de aplicação. Esses achados demonstram que a intervenção educativa foi eficaz em promover uma cobertura mais uniforme e adequada do produto. Além disso, houve um aumento no número de etapas corretamente seguidas durante a HM e uma redução no número de etapas esquecidas. O tempo de realização da técnica também aumentou, alinhando-se às recomendações da OMS e destacando a efetividade da intervenção com auxílio da gamificação.

A gamificação tem sido aplicada de forma crescente na educação permanente em saúde, com impactos positivos na aprendizagem de profissionais em diferentes áreas. Estratégias gamificadas em contextos clínicos e hospitalares demonstram aumento do engajamento, da retenção de informações e da adesão a protocolos, além de favorecer a aquisição de habilidades cognitivas, procedimentais e comportamentais. O número de publicações sobre gamificação em saúde tem crescido de forma expressiva, evidenciando o tema como emergente, com destaque para conceitos centrais, como aprendizagem baseada em jogos e *e-learning*. Dessa forma, a gamificação constitui uma abordagem em expansão, capaz de apoiar continuamente o desenvolvimento profissional e a aquisição de competências essenciais para a prática clínica segura^17^ ^-^ ^18^.

O jogo Hiji Sushi, desenvolvido pela Fiocruz, foi utilizado neste estudo como estratégia para o ensino da HM, demonstrando impacto inicial positivo na aprendizagem. Nesse sentido, a gamificação por meio de jogos tem se mostrado uma ferramenta inovadora na educação em saúde, promovendo o desenvolvimento cognitivo, a aquisição de informações e a mudança de comportamento. Foi realizado um estudo no qual verificam-se os benefícios significativos das intervenções gamificadas no ensino-aprendizagem, porém, não foram identificadas na literatura outras pesquisas que tenham utilizado esse jogo especificamente para o ensino da higiene das mãos, ressaltando a originalidade da abordagem^18^. 

Este estudo corrobora achados sobre a gamificação como estratégia eficaz no ensino da HM, avaliando a qualidade da técnica realizada, o tempo de execução, as etapas esquecidas e os locais mais frequentes iluminados pelo simulador fluorescente. Embora não seja voltado à prevenção e controle de IRAS, um estudo com o jogo *Oncologic Emergencies* demonstrou impacto positivo ao utilizar a abordagem gamificada na simulação de emergências oncológicas. A avaliação por meio do pré e pós-testes indicou melhora significativa no desempenho dos participantes, revelando que, além de consolidar conhecimentos e aprimorar o raciocínio clínico, a gamificação aumenta o interesse, a motivação e a satisfação no aprendizado^19^.

Sob outra perspectiva, um estudo investigou um jogo voltado para o desenvolvimento de habilidades diagnósticas em enfermagem, com o objetivo de proporcionar um ambiente controlado em que os alunos pudessem praticar análise clínica e tomada de decisão, simulando situações reais de trabalho. Os resultados indicaram que os participantes que utilizaram o jogo apresentaram maior segurança em cenários clínicos reais, evidenciando a relevância da gamificação na educação permanente dos profissionais^20^. Vale destacar que, embora a literatura seja ampla sobre intervenções educativas gamificadas em diversas áreas do conhecimento, a higiene das mãos, apesar de ser uma técnica simples e eficaz na prevenção e no controle de IRAS, pode se tornar mais complexa ao envolver aspectos individuais, do ambiente de trabalho, da equipe, da tarefa, do paciente, da organização e gestão^21^.

Foi possível observar que, ao participar do jogo, enfermeiros e técnicos de enfermagem refletiram sobre os locais preconizados que haviam sido esquecidos na etapa pré-intervenção. A OMS recomenda a técnica de HM em seis passos, seja com preparação alcoólica ou sabonete líquido, para garantir a redução da carga microbiana; contudo, uma revisão sistemática confirmou sua eficácia, mas destacou a heterogeneidade dos estudos e o alto risco de viés, indicando a necessidade de mais pesquisas para determinar a técnica mais eficaz e viável^22^. 

Nesse sentido, com o acesso ao jogo, o participante se encontra em um ambiente com todos os itens necessários para realização da HM, e só consegue avançar nas fases seguindo as seis etapas recomendadas pela OMS^14^ ^,^ ^22^. Esses aspectos corroboram a literatura, que aponta que o uso de jogos na educação em enfermagem, além de ter ganhado destaque nos últimos anos, também ressaltou tendências recentes e perspectivas futuras para o uso de intervenções gamificadas no processo de ensino-aprendizagem^20^.

O uso do simulador fluorescente mostrou distribuição insuficiente do produto nas mãos, com maior concentração nas palmas e nos polegares. Após a intervenção, a aplicação se tornou mais equilibrada, distribuída uniformemente. Um estudo-piloto utilizou imagens térmicas para avaliar a HM, com foco na uniformidade da distribuição da solução, refletindo a cobertura das áreas higienizadas. As imagens foram eficazes em identificar áreas negligenciadas, como espaços interdigitais, polpas digitais e polegares, frequentemente esquecidas e com alto risco de contaminação cruzada. Essa pesquisa revelou que até profissionais experientes cometeram falhas, e os autores destacam o potencial das tecnologias no treinamento e no monitoramento das práticas de higiene das mãos^23^. Por esse motivo, recomenda-se a incorporação dessas tecnologias como estratégia educativa para reforçar a conscientização, aperfeiçoar a técnica dos profissionais de enfermagem.

A intervenção educativa gamificada nesta pesquisa resultou em uma melhora significativa na execução correta da HM. Estratégias multimodais, quando combinadas a tecnologias digitais, ampliaram a acessibilidade e a flexibilidade no aprendizado. Métodos lúdicos e interativos demonstraram impacto positivo, promovendo mudanças comportamentais e favorecendo a prática da HM, especialmente quando adaptadas ao contexto profissional^24^. Um estudo quase-experimental, que avaliou a efetividade de uma intervenção educativa sobre a mesma temática em uma unidade de radiografia hospitalar, incluiu palestras, materiais visuais e dispensadores de álcool em gel. Após a intervenção, houve aumento de 28,9% para 51,4%, com melhora no conhecimento, na aceitação do produto utilizado e na percepção da necessidade de realizar a técnica após contato com áreas próximas ao paciente^25^.

Embora o conhecimento entre os profissionais de enfermagem seja de moderado a bom, destaca-se a necessidade de programas educativos contínuos que promovam a troca de experiências entre a equipe^26^. Em uma Unidade de Terapia Intensiva (UTI) de Cuiabá, Brasil, a realização da HM foi maior após a assistência, enquanto antes do contato com o paciente foi menor, e o uso de luvas impactou negativamente na promoção da técnica adequada^24^. Os achados desta pesquisa indicam que a implementação de intervenções educativas gamificadas pode aprimorar a qualidade da técnica, especialmente nos cinco momentos críticos preconizados pela OMS, além de contribuir para a segurança do paciente na prática clínica^14^.

A análise revelou um impacto no tempo despendido pelos enfermeiros e técnicos de enfermagem na realização da HM. Conforme as recomendações da OMS, o uso de preparações alcoólicas deve durar de 20 a 30 segundos, enquanto a higiene com água e sabonete líquido deve ocorrer entre 40 e 60 segundos. Embora haja diferenças no contexto de aplicação, é importante destacar que o uso de álcool em gel é recomendado quando há menor carga de sujidade, enquanto a água e o sabão são indicados para situações visíveis de sujeira^14^.

Neste estudo, o tempo de execução da HM apresentou conformidade com as recomendações vigentes, variando de 20 a 60 segundos, de acordo com a simulação do uso de preparações alcoólicas ou sabonete líquido, considerando as especificações dos produtos utilizados. Na avaliação pós-intervenção, todos os participantes permaneceram dentro do tempo recomendado - 20 a 30 segundos para a preparação alcoólica e 40 a 60 segundos para o sabonete líquido -, demonstrando maior alinhamento às diretrizes internacionais^2^ ^,^ ^14^ ^,^ ^27^. A execução correta da técnica dentro do tempo recomendado é fundamental para assegurar a cobertura completa de todas as superfícies das mãos, reforçando o potencial das intervenções educativas em promover o cumprimento adequado dessa prática^14^ ^,^ ^28^.

Esta pesquisa apresenta algumas limitações, incluindo a realização em um único hospital, o que restringe a generalização dos achados. Além disso, o delineamento quase-experimental do tipo antes e depois, com um curto intervalo de apenas uma hora entre as avaliações, impede a análise do impacto a médio e longo prazo da intervenção, influenciando a compreensão sobre a retenção de conhecimento e a manutenção das mudanças comportamentais. A ausência de seguimento longitudinal também impede analisar se os efeitos observados foram sustentáveis ao longo do tempo. A observação direta dos participantes pode ter sido influenciada pelo viés de Hawthorne; entretanto, esse risco foi minimizado por meio de rotinas padronizadas, permitindo que os profissionais retornassem às suas atividades habituais antes da reavaliação.

As contribuições para a prática clínica deste estudo reforçam a importância da incorporação de estratégias educacionais inovadoras, como a gamificação, para aprimorar a execução adequada da técnica de HM entre enfermeiros e técnicos de enfermagem. A implementação de abordagens lúdicas e interativas pode facilitar a aprendizagem, tornando o treinamento mais atrativo e eficaz, além de promover mudanças comportamentais sustentáveis. A adoção de tecnologias como simuladores fluorescentes e jogos digitais na educação em saúde contribui para a conscientização sobre a técnica adequada da equipe. Esses achados destacam a necessidade de integrar metodologias ativas aos programas de educação continuada, fortalecendo a prática baseada em evidências e qualificando o cuidado prestado aos pacientes.

## Conclusão

Conclui-se que a intervenção educativa gamificada foi efetiva na execução correta da técnica de higienização das mãos entre enfermeiros e técnicos de enfermagem em unidades de internação hospitalar. Entre os efeitos observados, destacaram-se a distribuição mais uniforme do produto fluorescente nas mãos, a redução de etapas negligenciadas e a melhoria na qualidade de execução da técnica, incluindo o cumprimento do tempo recomendado pela OMS, tanto com preparação alcoólica quanto com sabonete líquido. Esses achados evidenciam a efetividade da educação em saúde baseada em jogos na promoção de práticas mais seguras e eficazes, indicando potencial para contribuir na redução do risco de infecções relacionadas à assistência à saúde. Destaca-se que os achados têm caráter inicial e são necessários estudos com amostras maiores, em diferentes contextos e acompanhamento de longo prazo.

## Data Availability

Todos os dados gerados ou analisados durante este estudo estão incluídos neste artigo publicado.

# Translation: Efectividad de una intervención educativa gamificada sobre el lavado de manos entre profesionales de Enfermería: estudio cuasiexperimental

**DOI:** 10.1590/1518-8345.8067.4890

**Authors:** Maiara Luci Silva Costa, Márcio Medeiros, Fábio da Costa Carbogim, Thiago César Nascimento, André Luiz Silva Alvim

**Affiliations:** 1 Universidade Federal de Juiz de Fora, Juiz de Fora, MG, Brasil.; 2 Universidade Federal de Juiz de Fora, Faculdade de Enfermagem, Juiz de Fora, MG, Brasil.; 3 Universidade Federal de Juiz de Fora, Faculdade de Enfermagem, Departamento de Enfermagem Aplicada, Juiz de Fora, MG, Brasil.; 4 Universidade Federal de Juiz de Fora, Faculdade de Enfermagem, Departamento de Enfermagem Básica, Juiz de Fora, MG, Brasil.

**Keywords:** Desinfección de las Manos, Grupo de Enfermería, Gamificación, Educación en Salud, Educación, Control de Infecciones.

## Abstract

**Objetivo:**

evaluar la efectividad de una intervención educativa gamificada en la correcta ejecución de la técnica del lavado de manos entre enfermeros y técnicos de enfermería que trabajan en unidades de internación hospitalaria.

**Método:**

estudio cuasiexperimental del tipo antes y después, realizado con 45 profesionales de Enfermería. Los datos se recolectaron por medio de un formulario estructurado para observar la técnica en los períodos Pre- y Post-intervención. El análisis estadístico de los datos se realizó utilizando la prueba *Signed-Rank* de Wilcoxon y la prueba de McNemar.

**Resultados:**

durante el período Pre-intervención, el 61,3% de los técnicos y el 42,9% de los enfermeros no siguieron todos los pasos de la técnica; después de la gamificación, el 100% de los enfermeros y el 97,7% de los técnicos de enfermería implementaron los pasos correctos (*p*<0.001) y olvidaron menos etapas (*z*=-4,532; *p*<0,001; *r*=0,81). El tiempo dedicado a ejecutar la técnica también fue adecuado después de la intervención, siguiendo las recomendaciones de la Organización Mundial de la Salud (*z*=-2,441; *p*=0,015; *r*=0,36).

**Conclusión:**

la intervención educativa gamificada fue efectiva en la correcta ejecución de la técnica del Lavado de manos entre enfermeros y técnicos de enfermería, reduciendo la cantidad de errores y mejorando el cumplimiento de las pautas internacionales.

## Destacados

**(1)** La gamificación mejoró la adhesión al lavado de manos entre los profesionales de Enfermería. **(2)** La gamificación demostró ejercer un efecto positivo sobre el aprendizaje y la adhesión a la técnica. **(3)** La cantidad de etapas olvidadas durante el lavado de manos se redujo significativamente. **(4)** La distribución del producto sobre las manos fue más uniforme después de la intervención. **(5)** El tiempo dedicado a ejecutar la técnica fue adecuado después de la intervención educativa.

## Introducción

La transmisión de microorganismos a través de las manos de los profesionales de la salud es uno de los principales factores asociados a las infecciones relacionadas con la asistencia sanitaria (IRAS). Por ese motivo, además de ser de bajo costo y eficaz, el lavado de manos (LM) es una estrategia esencial para prevenir estos problemas en los lugares en los que se proporciona asistencia a los pacientes. La Organización Mundial de la Salud (OMS) recomienda cinco momentos específicos para reducir el riesgo de transmisión, a saber: antes de entrar en contacto con un paciente; antes de realizar procedimientos asépticos; después de estar expuesto a fluidos corporales; después de entrar en contacto con un paciente; y después de tocar superficies que se encuentran cerca de un paciente^1^.

Las IRAS son eventos adversos que se adquieren mientras se realizan procedimientos y/o internaciones hospitalarias o posteriormente a dichas actividades; además, imponen una significativa carga sobre los sistemas de salud y elevan los costos asistenciales. Estas infecciones constituyen uno de los problemas más frecuentes en el ámbito de los servicios de salud y un porcentaje sustancial de ellas es resultado de la acción de microorganismos multi-resistentes, lo que impone perjuicios a pacientes, visitas y profesionales de la salud por igual^2^.

Se estima que las IRAS son responsables por cerca de 3,5 millones de fallecimientos al año, además de representar costos superiores a mil millones de dólares para las arcas públicas. La incidencia de estos eventos adversos varía a nivel global; llegando aproximadamente al 7% en países desarrollados, al 8% en la Unión Europea y hasta al 15% en países en vías de desarrollo. En este contexto, implementar intervenciones por medio de sistemas multimodales demuestra ser altamente eficaz, con la posibilidad de evitar hasta 821.000 muertes y proporcionar un significativo retorno económico, estimándose que por cada dólar invertido en promover la práctica del LM se genera una ganancia de 24,6 dólares^3^.

El lavado correcto de manos es una medida clave no solo para prevenir la transmisión de infecciones IRAS sino también en la lucha contra la resistencia antimicrobiana, un problema en crecimiento a nivel mundial. Los profesionales de la salud (especialmente los de Enfermería) desempeñan un rol importante en el manejo de la asistencia provista al pie de la cama y en términos de implementar medidas de prevención en materia de salud. Esto implica seguir los protocolos específicos establecidos por las instituciones y promover la adhesión a las pautas recomendadas por la OMS y por la Agencia Nacional de Vigilancia Sanitaria (ANVISA), además de invertir en educación continua, garantizando que la totalidad del equipo esté actualizado con respecto a las mejores prácticas basadas en evidencias^3^ ^-^ ^4^.

Un consenso entre varios autores señala escasa adhesión a la práctica del LM entre profesionales de la salud. En un trabajo de investigación realizado en Brasil se observó que menos del 50% de los enfermeros se higienizan las manos correctamente y que la tasa es incluso más baja entre los técnicos de enfermería, puesto que no supera el 40%^5^ ^-^ ^6^. De manera similar, un estudio que realizó 292 observaciones durante una semana reveló que la adhesión de los auxiliares/técnicos de enfermería fue solo del 37%. Estos indicadores pueden considerarse como un problema multifactorial relacionado con la sobrecarga de trabajo, el estrés, un entorno físico no adecuado y la ubicación incorrecta de los lavabos y *dispensers* de solución a base de alcohol, además de factores conductuales no adecuados^7^.

La educación permanente en salud se ha utilizado ampliamente para mejorar la adhesión a la técnica del LM y su correcta ejecución. Pese a ello, todavía existen diversos desafíos, puesto que los índices frecuentemente retornan a los niveles iniciales después de implementar intervenciones tradicionales. Tecnologías innovadoras como videos interactivos, Realidad Virtual y gamificación surgen como herramientas promisorias para promover cambios sustentables en la práctica profesional^3^.

La gamificación se destaca por incorporar elementos lúdicos al proceso educativo; además, ha demostrado potencial para lograr el compromiso de los profesionales de Enfermería, mejorar la retención de conocimientos y refinar la ejecución de prácticas asistenciales^8^ ^-^ ^9^. Sin embargo, la principal brecha que pretende llenar este estudio es la escasez de trabajos de investigación dirigidos a enfermeros y técnicos de enfermería que trabajan de forma directa en la asistencia a los pacientes, puesto que la mayor parte de las investigaciones sobre gamificación se concentra en estudiantes de Enfermería^8^ ^,^ ^10^ ^-^ ^12^. Además, los trabajos existentes priorizan la adhesión a los cinco momentos del LM sin considerar la calidad técnica de su ejecución, incluido el uso adecuado de soluciones a base de alcohol o jabón líquido y el tiempo durante el cual se deben frotar las manos, lo que representa un factor limitante al momento de evaluar el nivel de cumplimiento con las pautas vigentes^8^ ^,^ ^10^ ^-^ ^11^.

Dada esta situación, el objetivo de este estudio es evaluar la efectividad de una intervención educativa gamificada en la correcta ejecución de la técnica del Lavado de manos entre enfermeros y técnicos de enfermería que trabajan en unidades de internación hospitalaria.

## Método

### Tipo de estudio

Estudio cuasiexperimental del tipo antes y después, con diseño no aleatorizado^13^. La investigación se realizó utilizando el mismo grupo de personas para los grupos Control y Experimental; eso implica que todos los participantes fueron sometidos a ambas condiciones de evaluación: los períodos Pre-intervención y Post-intervención.

### *Locus* del estudio

La investigación se realizó en un hospital universitario ubicado en la región de la *Zona da Mata Mineira*, Brasil, que atiende a pacientes del Sistema Único de Salud (SUS). El *locus* posee 137 camas de internación (incluidas nueve de Cuidados Intensivos) y desempeña un rol fundamental en la atención sanitaria a la comunidad local ofreciendo servicios de alta complejidad, además de ser un centro de capacitación para futuros profesionales de la salud. Haber elegido esta institución responde a motivos estratégicos, puesto que proporciona amplia interacción entre servicios de enseñanza, investigación y asistencia, enfocándose en la calidad de la atención provista a los pacientes de la región.

### Período

El estudio se realizó entre junio y diciembre de 2024.

### Población

La población estuvo compuesta por enfermeros y técnicos de enfermería que trabajan en las unidades de Cuidados Intensivos para adultos, Clínica Médica y Clínica Quirúrgica, el foco exploratorio de este estudio.

### Criterios de selección

Los criterios de inclusión fueron los siguientes: ser enfermeros o técnicos de enfermería con al menos un año de experiencia en la profesión. Este criterio referente al tiempo efectivo de ejercicio profesional se consideró importante debido a que implica suficiente experiencia práctica para evaluar la técnica del LM y familiaridad con los protocolos institucionales, redundando así en mayor precisión en los resultados. Se excluyó del estudio a profesionales con funciones administrativas y a residentes.

### Definición de la muestra

El cálculo muestral se realizó en el programa de *software* G*Power, sobre la base de la familia de pruebas t, específicamente para comparaciones bilaterales entre dos grupos. Se optó por definir un tamaño de efecto de 0,50, considerado medio, con error muestral de 0,05 y poder estadístico de 0,80. Se seleccionaron estas especificaciones para garantizar que el estudio tuviese una probabilidad adecuada de detectar diferencias significativas (en caso de existir alguna), minimizando la posibilidad de errores tipo II. Luego de incorporar estos parámetros, el resultado indicó que el tamaño total de la muestra requerido era de 34 participantes. Este valor se alcanzó con un poder efectivo de 0,8077, confirmando así que la muestra calculada era adecuada para los requerimientos del estudio. Sin embargo, a fin de mejorar la robustez de los resultados y compensar posibles pérdidas muestrales, se incluyó a 45 profesionales de Enfermería.

### Recolección de datos

El procedimiento para recolectar los datos estuvo a cargo de los propios investigadores, quienes se acercaron directamente al *locus* de investigación para invitar a enfermeros y técnicos de enfermería a participar en el estudio. El proceso se dividió en tres fases: Pre-intervención; Intervención educativa gamificada y Post-intervención.

*Fase 1: Pre-intervención*

Inicialmente, se indicó a los profesionales que realizaran la práctica del LM de la manera habitual. A fin de evaluar la cobertura y distribución de la técnica utilizada se aplicó la solución Optiglow^®^, que simula la presencia de microorganismos y se hace visible bajo luz fluorescente. La fluorescencia indica si el producto se aplicó correctamente, reflejando la correcta ejecución de la técnica sobre las áreas adecuadas, ya sea con la solución a base de alcohol o con jabón líquido. Se indicó a los participantes que ejecutaran la técnica como si estuvieran utilizando uno de esos productos, sin necesidad de aplicarlos.

*Fase 2: Intervención educativa gamificada*

Después de la evaluación inicial se condujo la intervención educativa en un ambiente reservado, minimizando las influencias externas. En la sesión de capacitación, la gamificación se utilizó como estrategia pedagógica para favorecer el aprendizaje de forma visual e interactiva. Se instruyó a los participantes individualmente o de a pares utilizando el juego Hiji Sushi, que se encuentra disponible en la plataforma Educare de la Fundación Oswaldo Cruz (Fiocruz). El juego simula los movimientos recomendados por la OMS para un correcto lavado de manos, presentando desafíos en los que los profesionales solo pueden avanzar a la siguiente etapa si implementan cada paso correctamente. El acceso a la plataforma fue de manera virtual por medio de un dispositivo electrónico del tipo *tablet* (un aparato portátil con pantalla táctil), lo que permitió que los participantes interactúen directamente con el contenido digital. A fin de garantizar homogeneidad en el uso de la herramienta, se capacitó previamente a todos los participantes sobre cómo manejarla, asegurando un nivel uniforme de familiaridad con la tecnología^14^ ^-^ ^15^.

Después de la intervención, se debatió lo siguiente con los participantes: el objetivo del juego Hiji Sushi; los cinco momentos recomendados por la OMS para el LM; y la importancia de esta práctica en la prevención de infecciones IRAS^14^ ^-^ ^15^. Se reservó un momento para aclarar dudas. Subsiguientemente, se liberó a los profesionales para que retomaran sus actividades. Se estableció un intervalo mínimo de una hora entre la intervención y la reevaluación; eso permitió que los profesionales retornaran a sus actividades habituales. El objetivo de este período fue reducir el efecto del híperfoco generado por la actividad educativa, permitiendo que los participantes retomaran sus rutinas y, posteriormente, fueran reevaluados en un contexto más cercano a su práctica cotidiana. De esta manera, se procuró llegar a un análisis más fidedigno sobre la retención de conocimientos y la incorporación de los movimientos recomendados para el LM.

*Fase 3: Post-intervención*

Después del intervalo, los participantes regresaron al ambiente reservado para volver a ser evaluados con respecto a la técnica del LM, utilizando los mismos procedimientos de la fase pre-intervención.

Desarrollado sobre la base de las recomendaciones de la OMS^14^, el instrumento de recolección de datos se utilizó para registrar diversa información sobre los participantes y la ejecución de la técnica del LM. Se evaluaron distintas variables, a saber: características sociodemográficas; cobertura y distribución del producto fluorescente sobre las manos y cantidad de etapas olvidadas al ejecutar la técnica, como enjabonar las palmas de las manos, masajear los espacios interdigitales, frotar el dorso de los dedos y masajear las yemas de los dedos y las uñas, entre otras. La ejecución de la técnica se clasificó categóricamente: Adecuada, cuando se cumplieron correctamente todas las etapas; y No eficaz, cuando se omitió cualquier etapa o se registraron errores en alguna de ellas.

El tiempo dedicado al LM se registró inicialmente con un cronómetro, para luego categorizarlo en franjas temporales específicas de acuerdo con las recomendaciones para cada tipo de técnica. El tiempo recomendado para la solución a base de alcohol es de 20 a 30 segundos, mientras que en el caso del jabón líquido es de 40 a 60 segundos^14^. En consecuencia, tiempos inferiores a los recomendados, superiores al límite máximo o entre 30 y 39 segundos se consideraron no adecuados, puesto que no cumplían con los estándares establecidos para que la técnica sea efectiva. Para el análisis estadístico, el tiempo se trató como variable continua en la comparación entre los períodos Pre- y Post-intervención.

### Procesamiento y análisis de los datos

Los datos recolectados se analizaron por medio de distintas pruebas estadísticas. Inicialmente, se empleó estadística descriptiva para caracterizar las variables sociodemográficas. La normalidad de los datos se evaluó utilizando la prueba de Shapiro-Wilk. En caso de no detectarse distribución normal de los datos, se optó por usar la prueba *Signed-Rank* de Wilcoxon para los análisis. También se consideró el tamaño del efecto, interpretado de acuerdo con los siguientes criterios: valores superiores a 0,2 indican efecto pequeño, superiores a 0,5 representan efecto medio y superiores a 0,8 reflejan efecto grande^16^. Para evaluar cambios en la correcta ejecución de la técnica del LM a lo largo del estudio se aplicó el análisis de McNemar, en el que se verificaron alteraciones en las respuestas binarias (Sí o No) antes y después de la intervención. Todos los análisis estadísticos se realizaron utilizando el programa de *software Statistical Package for the Social Sciences*(SPSS), versión 21.

### Aspectos éticos

Este estudio fue aprobado por un Comité de Ética en Investigación (*Comitê de Ética em Pesquisa*, CEP) con el siguiente número de opinión: 6.600.099. Todos los participantes leyeron y firmaron un Formulario de Consentimiento Libre e Informado (FCLE).

## Resultados

La muestra estuvo compuesta por 45 profesionales: 31 (68,9%) técnicos de enfermería y 14 (31,1%) enfermeros. En cuanto al sexo de los participantes, 31 (68,9%) eran mujeres y 14 (31,1) eran hombres. En relación al color de piel, 21 (46,7%) se identificaron como de piel morena, 16 (35,6%) como de piel blanca y 8 (17,8%) como de piel negra. En referencia a los sectores de trabajo, 19 (42,2%) se desempeñaban en la Unidad Quirúrgica, 13 (28,9%) lo hacían en la Unidad de Cuidados Intensivos y 13 (28,9%) en la Unidad de Clínica Médica. Con respecto a la capacitación en el lavado de manos, 35 (77,8%) indicaron ya haber participado en alguna sesión, mientras que 10 (22,2%) nunca se habían sometido a dicha capacitación. Entre los sujetos que recibieron capacitación, 16 (35,6%) la habían cursado hacía más 1 año, 14 (31,1%) hacía menos de 6 meses y 6 (13,3%) entre 6 meses y 1 año atrás; además, 9 (20%) de los profesionales indicaron no poseer ninguna formación previa sobre el tema.

Durante el período Pre-intervención, el análisis reveló que la palma de la mano izquierda fue el área que la solución fluorescente iluminó con mayor frecuencia, con 19 registros (61,3%), seguida por la palma de la mano derecha con 16 (51,6%). Las yemas de los dedos de las manos izquierda y derecha se observaron en 15 (48,4%) y en 13 (41,9%) casos, respectivamente, mientras que los pulgares derecho e izquierdo figuraron en 12 (38,7%) y 11 (35,5%) registros. Entre los enfermeros, el dorso de la mano derecha fue la región en la que más se concentró el producto, con nueve casos (64,3%), seguido por el dorso de la mano izquierda con siete (50,0%) y la palma de la mano derecha con seis casos (42,9%). En el simulador fluorescente, las áreas más iluminadas indican concentración del producto; es decir, regiones que recibieron más atención durante el LM, mientras que las áreas menos iluminadas reflejan puntos desatendidos. Durante el período Post-intervención, la distribución del producto fue uniforme en ambas categorías, indicando una mejora en la ejecución de la técnica. Sin embargo, también hubo registros de producto concentrado en el pulgar derecho entre los técnicos, con 10 (32,3%) observaciones, seguidos por las palmas de las manos derecha e izquierda con nueve (29,0%) y ocho (25,8%) casos, respectivamente. Entre los enfermeros solo se observaron cuatro (28,6%) registros en el pulgar de la mano izquierda.

En este contexto, los resultados indican que la intervención educativa ejerció un efecto positivo en relación a reducir la cantidad de áreas con concentración de producto fluorescente en puntos específicos, siendo menor en la etapa Post-intervención (*M*=1,46; *DE*=1,15) en comparación con la Pre-intervención (*M*=3,24; *DE*=1,62), con *z*=-5,450; *p*<0,001; y tamaño de efecto grande (*r*=0,81).

Los resultados demostraron un cambio estadísticamente significativo (*p*<0,001); eso indica que la cantidad de sujetos que siguieron las etapas conforme a lo recomendado por la OMS fue mayor después de la intervención. En la Pre-intervención, 25 (55,6%) de los profesionales de Enfermería no siguieron todos los pasos recomendados: 19 (61,3%) técnicos y 6 (42,9%) enfermeros. Se observó una mejora significativa después de la intervención gamificada: 44 (97,7%) de los profesionales comenzaron a ejecutar la técnica correctamente, especialmente los 14 (100%) enfermeros; apenas 1 (2,3%) técnico de enfermería siguió sin realizarla de forma adecuada.

La cantidad de etapas olvidadas durante el procedimiento de LM se evaluó antes y después de la intervención educativa entre las categorías profesionales. Los resultados demostraron que se olvidaron menos etapas en el período Post-intervención (*M*=0,02; *DE*=0,14) en comparación con la Pre-intervención (*M*=0,80; *DE*=0,84), con *z*=-4,532; *p*<0,001; y tamaño de efecto medio (*r*=0,81) (Tabla 1).

**Tabla 1:** Etapas que los profesionales de Enfermería olvidaron durante el lavado de manos, antes y después de la intervención educativa. Juiz de Fora, MG, Brasil, 2025

Etapas para LM*, de acuerdo con la OMS^†^(15)	Descripción de las etapas	Pre-intervención^‡^	Post-intervención
		n (%)	n (%)
	Enjabonar las palmas de las manos, masajeándolas una con otra	05 (12,8)	-
	Frotar la palma de la mano derecha contra el dorso de la izquierda entrelazando los dedos, y viceversa	02 (5,1)	-
	Entrelazar los dedos y masajear los espacios interdigitales	09 (33,0)	-
	Frotar el dorso de los dedos de una mano con la palma de la otra mano, sujetando los dedos y con un movimiento de vaivén, y viceversa	01 (5,9)	-
	Frotar el pulgar izquierdo con la ayuda de la palma de la mano derecha y con un movimiento circular, y viceversa	07 (27,9)	-
	Masajear las yemas de los dedos y las uñas de la mano derecha contra la palma de mano izquierda con un movimiento circular, y viceversa	12 (37,2)	01 (2,2)
	No olvidaron ninguna etapa	20 (77,9)	44 (97,8)

*LM = Lavado de manos; ^†^OMS = Organización Mundial de la Salud; ^‡^Se observó más de una etapa olvidada por cada profesional de Enfermería evaluado

Se destaca que el tiempo dedicado al LM después de la intervención entre los enfermeros y técnicos de enfermería fue más prolongado, de acuerdo con las pautas propuestas por la OMS (Tabla 2). La intervención educativa demostró ser eficaz, puesto que los profesionales ejecutaron la técnica en el tiempo adecuado durante el período Post-intervención (*M*=3,55; *DE*=0,50) en comparación con la etapa Pre-intervención (*M*=3,18; *DE*=0,80), con *z*=-2,441; *p*=0,015; y tamaño de efecto pequeño (*r*=0,36).

**Tabla 2:** Evaluación del tiempo dedicado por los profesionales de Enfermería al lavado de manos, de acuerdo con lo recomendado por la OMS* y para los períodos Pre- y Post-intervención (n = 45). Juiz de Fora, MG, Brasil, 2025

Tiempo	Pre-intervención n (%)			Post-intervención n (%)	
	Téc. Enf.^†^	Enf.^‡^		Téc. Enf.^†^	Enf.^‡^
Menos de 20 segundos	01 (3,2)	0 (0)		-	-
Entre 20 y 30 segundos^§^	07 (22,6)	01 (7,10)		11 (35,5)	9 (64,3)
Entre 40 y 60 segundos^ǀǀ^	14 (45,2)	04 (28,6)		20 (64,5)	5 (35,7)
Más de 60 segundos	09 (29,0)	09 (64,3)		-	-

*Organización Mundial de la Salud; ^†^Técnicos de Enfermería; ^‡^Enfermeros; ^§^Tiempo adecuado recomendado por la OMS para higienizar las manos con solución a base de alcohol; ^ǀǀ^Tiempo adecuado recomendado por la OMS para higienizar las manos con jabón líquido

## Discusión

En este estudio se identificó que la aplicación del producto sobre las manos de los profesionales de Enfermería era irregular antes de la intervención, con mayor concentración en las regiones de las palmas, las yemas y los pulgares. Se verificó una distribución uniforme después de la intervención, con una significativa reducción en la cantidad de áreas que inicialmente presentaban mayor densidad de producto aplicado. Estos hallazgos demuestran que la intervención educativa fue eficaz en términos de promover una cobertura más uniforme y adecuada del producto. Además, se registró un aumento en la cantidad de etapas que se siguieron correctamente durante el LM y una reducción en el número de etapas olvidadas. El tiempo dedicado a la técnica también se prolongó, alineándose con las recomendaciones de la OMS y destacando la efectividad de la intervención con el auxilio de la gamificación.

La gamificación se ha venido aplicando cada vez más en la educación permanente en salud, con efectos positivos sobre el aprendizaje de profesionales de distintas áreas. Además de favorecer la adquisición de habilidades cognitivas, procedimentales y conductuales, las estrategias gamificadas en contextos clínicos y hospitalarios demuestran mejorar el compromiso, la retención de información y la adhesión a los protocolos. La cantidad de materiales publicados sobre la gamificación en salud ha crecido de manera significativa, evidenciando que se trata de un tema emergente con destaque en conceptos centrales como el aprendizaje basado en juegos e *e-learning*. En consecuencia, la gamificación constituye un enfoque en expansión capaz de auxiliar continuamente el desarrollo profesional y la adquisición de competencias esenciales para una práctica clínica segura^17^ ^-^ ^18^.

Desarrollado por la Fundación Fiocruz, el juego Hiji Sushi se utilizó en este estudio como una estrategia para enseñar la técnica del LM, demostrando un efecto inicial positivo sobre el aprendizaje. En este sentido, la gamificación por medio de juegos ha demostrado ser una herramienta innovadora en educación sanitaria, promoviendo el desarrollo cognitivo, la adquisición de información y cambios conductuales. Un estudio señala los significativos beneficios de las intervenciones gamificadas en el proceso de enseñanza-aprendizaje; sin embargo, no se encontraron otros trabajos de investigación en la literatura que hayan empleado este juego específicamente para enseñar la técnica del lavado de manos, resaltando así la originalidad de este enfoque^18^.

Este estudio corrobora hallazgos sobre la gamificación como estrategia eficaz para enseñar la técnica del LM, evaluando la calidad de la técnica aplicada, el tiempo dedicado a su ejecución, las etapas olvidadas y las áreas que el simulador fluorescente iluminó con mayor frecuencia. Aunque no estuvo dirigido a la prevención y el control de infecciones IRAS, un estudio realizado con el juego *Oncologic Emergencies* demostró un efecto positivo al utilizar el enfoque gamificado en la simulación de emergencias oncológicas. La evaluación por medio de los pre- y post-tests indicó una mejora significativa en el desempeño de los participantes, revelando que, además de consolidar conocimientos y mejorar el raciocinio clínico, la gamificación aumenta los niveles de interés, motivación y satisfacción por aprender^19^.

Desde otra perspectiva, un estudio investigó un juego dirigido a desarrollar habilidades diagnósticas en Enfermería con el objetivo de proporcionar un entorno controlado en el que los alumnos pudieran practicar análisis clínicos y procesos para tomar decisiones, simulando situaciones de trabajo reales. Los resultados indicaron que los participantes que utilizaron el juego se sintieron más seguros en situaciones clínicas reales, evidenciando así la relevancia de la gamificación y de la educación continua de los profesionales^20^. Cabe destacar que, aunque existe vasta literatura sobre intervenciones educativas gamificadas en diversas áreas del conocimiento y a pesar de que el lavado de manos es una técnica simple y efectiva para prevenir y controlar infecciones IRAS, puede volverse más compleja si se incluyen aspectos individuales, del entorno de trabajo, de los equipos de trabajo, de las tareas, de los pacientes, de la organización y de la gestión^21^.

Se pudo observar que, al participar en el juego, los enfermeros y técnicos de enfermería reflexionaron sobre las áreas recomendadas que habían olvidado en la etapa Pre-intervención. Ya sea con la solución a base de alcohol o con jabón líquido, la OMS recomienda una técnica de LM en seis pasos para garantizar una reducción en la carga microbiana; aunque una revisión sistemática confirmó su eficacia, destacó la heterogeneidad de los estudios y el elevado riesgo de sesgo, indicando que se requieren más estudios para determinar cuál es la técnica más eficaz y viable^22^.

En este sentido, al acceder al juego los participantes se encuentran en un ambiente con todos los elementos necesarios para ejecutar la técnica del LM y solo pueden avanzar a la fase siguiente si cumplen las seis etapas recomendadas por la OMS^14^ ^,^ ^22^. Estos aspectos se condicen con la literatura, que señala que, además de haber ganado notoriedad en los últimos años, utilizar juegos en la educación de Enfermería también destacó tendencias recientes y perspectivas a futuro para emplear intervenciones gamificadas en el proceso de enseñanza-aprendizaje^20^.

Al utilizarse el simulador fluorescente, fue posible detectar una distribución insuficiente del producto sobre las manos, con mayor concentración en las palmas y los pulgares. La aplicación fue más equilibrada y el producto se distribuyó mejor después de la intervención. En un estudio piloto se utilizaron imágenes térmicas para evaluar el LM, enfocándose en la uniformidad con la cual se distribuía la solución y reflejando la cobertura de las áreas higienizadas. Las imágenes resultaron eficaces para identificar áreas desatendidas (como los espacios interdigitales, las yemas de los dedos y los pulgares), olvidadas con frecuencia y con alto riesgo de contaminación cruzada. En este trabajo de investigación se detectó que incluso los profesionales más experimentados cometieron errores; además, los autores destacan el potencial de las tecnologías en materia de capacitación y supervisión de las prácticas de lavado de manos^23^. Por ese motivo, se recomienda incorporar estas tecnologías como una estrategia educativa para reforzar la concientización y refinar la técnica de los profesionales de Enfermería.

La intervención educativa gamificada descrita en este trabajo de investigación generó una significativa mejora en la correcta ejecución de la técnica del LM. Cuando se combinaron con tecnologías digitales, las estrategias multimodales ampliaron la accesibilidad y flexibilidad del aprendizaje. Los métodos lúdicos e interactivos demostraron ejercer un efecto positivo, promoviendo cambios conductuales y favoreciendo la práctica del LM, especialmente cuando se la adaptó al contexto profesional^24^. Un estudio cuasiexperimental que evaluó la efectividad de una intervención educativa sobre la misma temática en una unidad de Radiografía hospitalaria incluyó clases expositivas, materiales visuales y *dispensers* de alcohol en gel. Después de la intervención se registró un aumento del 28,9% al 51,4% en el nivel de conocimientos, la aceptación del producto utilizado y la percepción de que es necesario aplicar la técnica después de entrar en contacto con áreas próximas a un paciente^25^.

Aunque el nivel de conocimientos entre los profesionales de Enfermería es de moderado a bueno, se destaca la necesidad de implementar programas de educación continua que promuevan el intercambio de experiencias entre los integrantes de los equipos de trabajo^26^. En una Unidad de Cuidados Intensivos (UCI) de Cuiabá, Brasil, la técnica del LM se ejecutó más frecuentemente después de asistir a los pacientes, mientras que se realizó en menor medida antes de entrar en contacto con los pacientes; además, usar guantes ejerció un efecto negativo en materia de promover la técnica adecuada^24^. Los hallazgos de este estudio indican que implementar intervenciones educativas gamificadas puede mejorar la calidad de la técnica, especialmente en los cinco momentos críticos recomendados por la OMS, además de contribuir a la seguridad del paciente en la práctica clínica^14^.

El análisis reveló un efecto sobre el tiempo que dedican los enfermeros y técnicos de enfermería a la técnica de LM. De acuerdo con las recomendaciones de la OMS, las soluciones a base de alcohol deben usarse durante 20 a 30 segundos, mientras que el lavado de manos con agua y jabón líquido debe durar entre 40 y 60 segundos. Aunque haya diferencias en el contexto de aplicación, es importante destacar que se recomienda utilizar alcohol en gel cuando el nivel de suciedad es más bajo, mientras que se indica agua y jabón para situaciones de suciedad que pueden notarse a simple vista^14^.

En este estudio, el tiempo dedicado a ejecutar la técnica del LM cumplió con las recomendaciones vigentes, variando de 20 a 60 segundos de acuerdo con la simulación del uso de soluciones a base de alcohol o con jabón líquido y considerando las especificaciones de los productos utilizados. En la evaluación Post-intervención, todos los participantes permanecieron dentro del tiempo recomendado (de 20 a 30 segundos para la solución a base de alcohol y de 40 a 60 segundos en el caso del jabón líquido), demostrando condecirse más con las pautas internacionales^2^ ^,^ ^14^ ^,^ ^27^. Ejecutar la técnica correctamente dentro del tiempo recomendado es fundamental para garantizar una cobertura completa de todas las superficies de las manos, reforzando el potencial de las intervenciones educativas al momento de promover el correcto cumplimiento de esta práctica^14^ ^,^ ^28^.

Este trabajo de investigación presenta algunas limitaciones, como el hecho de haber sido realizado en un solo hospital, lo que restringe la generalización de los hallazgos. Además, el diseño cuasiexperimental del tipo antes y después con un breve intervalo de apenas una hora entre las evaluaciones impide apreciar el efecto de la intervención a mediano y largo plazo; eso influencia lo que puede saberse sobre la retención de conocimientos y la sustentabilidad de los cambios conductuales. No haber implementado un seguimiento longitudinal también impide analizar si los efectos observados se mantuvieron en el tiempo. La observación directa de los participantes pudo haberse visto influenciada por el efecto Hawthorne; no obstante, este riesgo se minimizó por medio de rutinas estandarizadas, permitiendo que los profesionales reanudaran sus actividades habituales antes de la reevaluación.

Los aportes de este estudio para la práctica clínica refuerzan la importancia de incorporar estrategias educativas innovadoras como la gamificación para mejorar la correcta ejecución de la técnica del LM entre enfermeros y técnicos de enfermería. Implementar enfoques lúdicos e interactivos puede facilitar el aprendizaje, haciendo que la capacitación sea más atrayente y eficaz, además de promover cambios conductuales sustentables. Adoptar tecnologías como simuladores fluorescentes y juegos digitales en la educación para la salud ayuda a generar conciencia sobre la técnica adecuada que deben implementar los profesionales. Estos hallazgos destacan la necesidad de integrar metodologías activas a los programas de educación continua, fortaleciendo las prácticas basadas en evidencias y cualificando la atención provista a los pacientes.

## Conclusión

Se concluyó que la intervención educativa gamificada fue efectiva en la correcta ejecución de la técnica del Lavado de manos entre enfermeros y técnicos de enfermería que trabajan en unidades de internación hospitalaria. Entre los efectos observados se destacaron los siguientes: distribución más uniforme del producto fluorescente sobre las manos; menos etapas desatendidas y mejoras en la calidad de ejecución de la técnica, incluido cumplir con los tiempos recomendados por la Organización Mundial de la Salud, tanto con la solución a base de alcohol como con el jabón líquido. Estos hallazgos evidencian la efectividad de la educación sanitaria basada en juegos al momento de promover prácticas más seguras y eficaces, indicando su potencial para ayudar a reducir el riesgo de infecciones relacionadas con la atención sanitaria. Se destaca que los hallazgos son de carácter inicial y que es necesario realizar estudios con muestras más grandes, en distintos contextos y con seguimiento a largo plazo.

## Data Availability

Todos los datos generados o analizados durante este estudio están incluidos en este artículo publicado.
